# Rapid room-temperature synthesis of ultrasmall cubic Mg–Mn spinel cathode materials for rechargeable Mg-ion batteries†

**DOI:** 10.1039/c9ra08626a

**Published:** 2019-11-08

**Authors:** Hiroaki Kobayashi, Kazuya Yamaguchi, Itaru Honma

**Affiliations:** Institute of Multidisciplinary Research for Advanced Materials, Tohoku University 2-1-1 Katahira, Aoba-ku Sendai Miyagi 980-8577 Japan h.kobayashi@tohoku.ac.jp; Department of Applied Chemistry, School of Engineering, The University of Tokyo 7-3-1 Hongo, Bunkyo-ku Tokyo 113-8656 Japan

## Abstract

Reducing the particle size of cathode materials is effective to improve the rate capability of Mg-ion batteries. In this study, ultrasmall cubic Mg–Mn spinel oxide nanoparticles approximately 5 nm in size were successfully synthesized *via* an alcohol reduction process within 30 min at room temperature. Though the particles aggregated to form large secondary particles, the aggregation could be suppressed by covering the particles with graphene. The composite exhibited a specific capacity of 230 mA h g^−1^, and could be cycled more than 100 times without any large capacity loss even at a moderate current density with the Mg(ClO_4_)_2_/CH_3_CN electrolyte.

## Abstract 2

An ultrasmall cubic Mg–Mn spinel obtained by an alcohol reduction process is demonstrated as a cathode candidate for rechargeable Mg-ion batteries.

## Graphical abstract

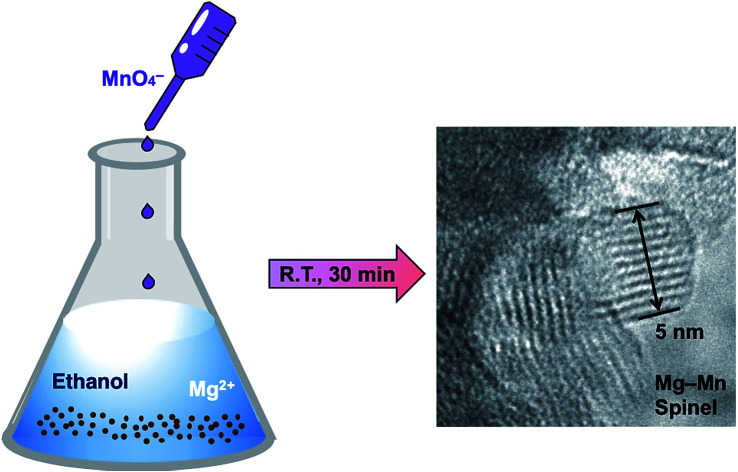

## Introduction

High energy and high density rechargeable batteries are indispensable for widespread portable electronic devices and the spreading of electric vehicles. Currently, Li-ion batteries have been widely utilized for these high-power applications,^1^ but to meet increasing requirements to enhance their energy densities, it is necessary to develop novel energy storage systems, such as Li–air,^2,3^ Li–S,^2,4^ and multivalent-ion batteries.^5^ Rechargeable Mg-ion batteries have gained much attention as promising alternatives to Li-ion batteries due to the high natural abundance, high volumetric energy density, and no dendrite formation of the Mg metal anode.^6–9^ While Mg-ion batteries are nowadays progressing rapidly, their practical use is still hampered by problems related to both cathode materials and electrolytes. One of the crucial problems is the very low rate capability at the cathodes due to the slow diffusion of Mg^2+^ ions in solids.^10^ Therefore, most reported Mg-ion batteries work at low current densities or at high temperature. This problem could be solved using water-based or water-added electrolytes, but they are incompatible with Mg metal anodes.^6^

Among various cathode candidates for Mg-ion batteries, spinel oxides have a high redox potential and a relatively low diffusivity.^11^ Especially in the common electrochemical window of electrolytes (<3.5 V *vs.* Mg/Mg^2+^), MgMn_2_O_4_ can exhibit a high theoretical capacity of 540 mA h g^−1^ using both Mn^3+^/Mn^4+^ and Mn^3+^/Mn^2+^ redox according to the following reactions:

1 MgMn_2_O_4_ ⇄ Mn_2_O_4_ (*λ*-MnO_2_) + Mg^2+^ + 2e^−^

2 MgMn_2_O_4_ + Mg^2+^ + 2e^−^ ⇄ Mg_2_Mn_2_O_4_ (rock-salt)

In eqn (1), extraction/insertion of Mg in a tetrahedral site proceeds with holding the host spinel structure, while a transformation reaction between MgMn_2_O_4_ spinel and Mg_2_Mn_2_O_4_ rock-salt proceeds *via* push-out process in eqn (2).^12^ Recently, MgMn_2_O_4_ with particle sizes of 11–200 nm were synthesized by various processes such as sol–gel and co-precipitation methods, and their cathode performances were investigated.^12–23^ Some reports demonstrated high specific capacities, but so far, there are no reports where high capacity could be obtained at high rates with an anhydrous electrolyte under ambient temperature. Since decreasing the particle size is one of the most effective ways to enhance the specific capacity and the rate capability for suppressing the slow Mg^2+^ diffusion in solids,^24^ alternative synthetic methods that enable nanoparticles below 10 nm are required for achieving a breakthrough in such cathodes.

In addition, the MgMn_2_O_4_ has a tetragonal spinel structure due to the Jahn–Teller effect of Mn^3+^ ions,^25^ while *λ*-MnO_2_ and Mg_2_Mn_2_O_4_ rock-salt are cubic phase. The above redox reactions should exhibit large polarizations because of the less reversible tetragonal–cubic phase transitions, hence a suppression of the lattice distortion of MgMn_2_O_4_ is necessary for improving its cathode performance. Feng *et al.* reported thin films of cubic-MgMn_2_O_4_ spinel obtained by PLD methods exhibited higher cathode performances than those for tetragonal-MgMn_2_O_4_ thin films.^17^ Although a cubic MgMn_2_O_4_ phase is known as a meta-stable phase,^26^ this phase is only obtained at high-temperature (>950 °C)^27,28^ or high-pressure (>15.6 GPa)^29^ in bulk.

Here we demonstrate high cathode performances of ultrasmall cubic Mg–Mn spinels synthesized *via* an alcohol reduction process. The process is commonly applied for synthesizing metal nanoparticles as a wet-process, and recently our group applied it for various Mn oxides and showed high catalytic activity for aerobic oxidation reactions.^30–32^ We also reported a high-rate capability of LiMn_2_O_4_ nanospinels in a Li-ion battery.^30^ In this study, an Mg–Mn spinel oxide (MMO) with approximately 5 nm was prepared by reduction of MnO_4_^−^ in an anhydrous MgCl_2_ solution in ethanol within 30 min at room temperature, and its cathode performances were investigated.

## Results and discussion

**Fig. 1:**
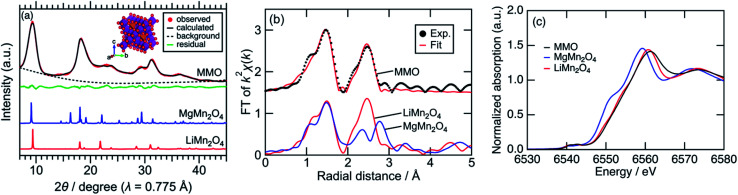
(a) Synchrotron XRD patterns with the fitting curve by the Rietveld refinement and the schematic illustration for cubic Mg_0.81_Mn_2_O_4_, (b) p-RDF obtained from the FT of *k*^2^-weighted Mn K-edge EXAFS spectra with the fitting curve by the first and second coordination shell analysis, and (c) Mn K-edge XANES spectra.

Fig. 1a shows synchrotron powder XRD patterns of MMO, MgMn_2_O_4_ spinel prepared by a sol–gel method,^19^ and LiMn_2_O_4_ spinel. Peaks of MMO were very broad, indicating the formation of nanocrystals as described later. The XRD pattern of MMO was similar to that of cubic LiMn_2_O_4_ spinel rather than that of tetragonal MgMn_2_O_4_ spinel. In the pseudo radial distribution functions (p-RDF) obtained by a Fourier transform (FT) of *k*^2^-weighted Mn K-edge EXAFS spectra (Fig. 1b), MMO and LiMn_2_O_4_ had a single peak at 2.48 Å attributed to isotropic Mn–Mn pairs, whereas MgMn_2_O_4_ had two peaks at 2.36 Å and 2.76 Å attributed to anisotropic Mn–Mn pairs.^33^ The EXAFS spectrum of MMO was well fitted using the first coordination shell consisting of single Mn–O paths (1.891(7) Å) and the second coordination shell consisting of single Mn–Mn paths (2.88(1) Å), suggesting that no Jahn–Teller distortion occurred in MMO to form a cubic phase (Table 1). From the result of ICP elemental analyses of Mg/Mn = 0.40(3) (mol mol^−1^), the formula of MMO can be described as Mg_0.80(6)_Mn_2_O_4_ or (Mg_0.86(4)_Mn_0.14(4)_)Mn_2_O_4_ with the average valence state of Mn^3.20(6)+^ or Mn^2.93(7)+^, respectively. In the former structure, vacancies in cation sites form, while the latter structure is a solid-solution of MgMn_2_O_4_ and Mn_3_O_4_. Mn K-edge XANES spectra shown in Fig. 1c supported the former structure. The Mn K-edge energy was higher than that of MgMn_2_O_4_ and was similar to that of LiMn_2_O_4_, supporting a higher valence state of Mn in MMO than trivalent. The XRD pattern in Fig. 1a was successfully fitted to Mg_0.81(3)_Mn_2_O_4_ with the *Fd*3̄*m* space group by the Rietveld refinement (Table 2), though the error of refined parameters should become large with such broad XRD patterns. According to the result, and cation-mixing between Mg and Mn is not observed^34^ and only vacancy in Mg-site formed.^35^ The estimated formula is consistent with the ICP elemental analyses, and estimated bond length of Mn–O and Mn–Mn are 1.988(5) Å and 2.878(1) Å, respectively, supporting the result of EXAFS analysis. The estimated structure is closely similar to the meta-stable cubic-MgMn_2_O_4_ phase^17^ or Mg_*y*_Mn_2_O_4_ solid solution phase between *λ*-MnO_2_ and MgMn_2_O_4_.^36^ Since the reduction reaction of MnO_4_^−^ ion proceeds rapidly to form nanoparticles without heating-up, once a meta-stable phase formed, the phase transition to more stable phase hardly proceed. The cubic phase is obtained probably due to the rapid nucleation of MMO particles at room temperature.

**Table 1:** Results of EXAFS analysis of MMO^a^

	Coordination number	Distance/Å	Debye–Waller factor/Å^2^
First coordination of Mn–O	1.0(2)	1.981(7)	0.003(1)
Second coordination of Mn–Mn	0.7(2)	2.88(1)	0.005(2)

a *R* = 1.97%.

**Table 2:** Results of the Rietveld refinement

Phase	Space group	Lattice parameters (Å)	Atom	Site	Occupancy (*g*) and atomic coordination
MMO^a^	*Fd*3̄*m*	*a* = 8.141(4)	Mg	8*b*	*g* = 0.81(3)
			Mn	16*c*	*g* = 1.00(1)
			O	32*e*	*x* = 0.2440(6)

a Partial profile relaxation was applied to 400, 440, and 444 reflection peaks. *R*_wp_ = 2.81%, *R*_p_ = 2.31%, *R*_e_ = 1.65%, *S* = 1.70.

In our previous work, only todorokite-type Mg–Mn binary oxide (OMS-1) was obtained when a hydrous MgCl_2_·6H_2_O dissolved in 2-propanol was used as a Mg-source.^31^ In the solution, Mg^2+^ ions were coordinated by water due to their strong Lewis acidic behaviour, and the aqueous complexes were introduced into manganese oxides without dehydration to form OMS-1. In the present work, we found that primary alcohols (*e.g.*, ethanol) have enough solubility of an anhydrous MgCl_2_ and reducing ability of permanganates instead of secondary alcohols. In the anhydrous condition, Mg^2+^ ions are solvated by ethanol without strong coordination, and are introduced into manganese oxides with desolvation to form a spinel-type Mg–Mn binary oxide. Such difference of products by the amount of water in reaction solutions is also observed in the case of Li–Mn and Co–Mn binary oxides.^31^

**Fig. 2:**
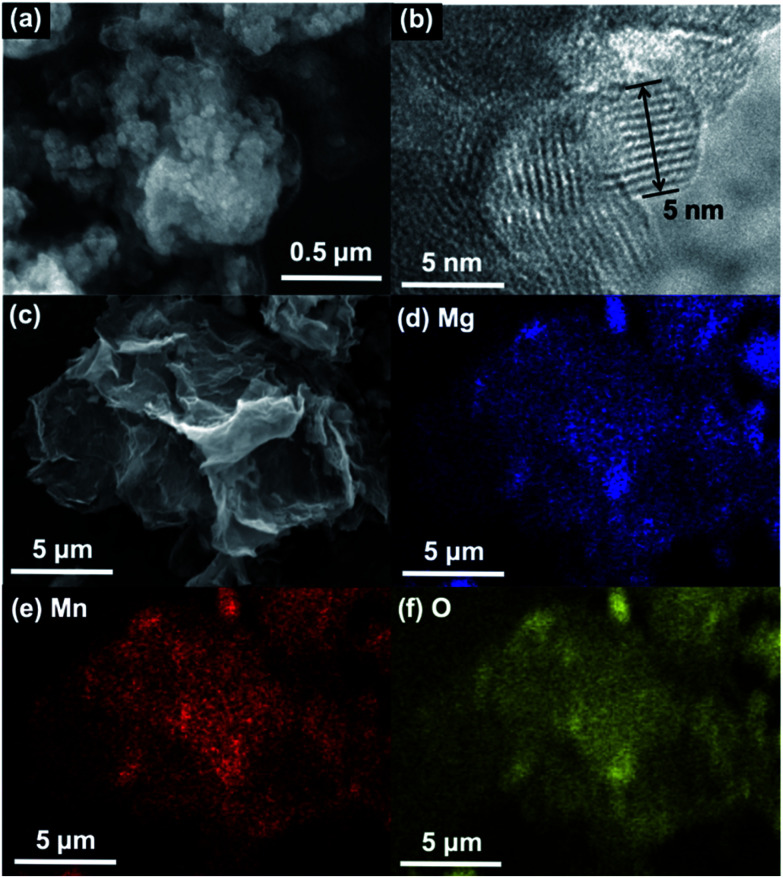
(a) A SEM and (b) a TEM image of MMO particles. (c) A SEM image and (d–f) EDX mappings of MMO–G composites.

Next, surface morphology of MMO particles were investigated. Fig. 2a and b show SEM and TEM images of MMO particles, respectively. These micrographs displayed submicron aggregates of approximately 5 nm nanoparticles with a clear lattice fringe spacing of 0.47 nm, which could be attributed to the 111 plane of the cubic spinel structure. The obtained MMO nanoparticles have much smaller crystallites than previously reported MgMn_2_O_4_ nanoparticles (>10 nm) synthesized by a sol–gel method.^16,18–23^ These ultrasmall particles are likely to have been obtained using this wet-process by suppressing the dissolution–recrystallization at ambient temperature and in an anhydrous solvent, while the sol–gel method contains a calcination step at which crystal growth should proceed. A high Brunauer–Emmett–Teller (BET) surface area of 151 m^2^ g^−1^ was also obtained, validating the formation of ultrasmall nanoparticles with below 10 nm in size. However, these large aggregates formed in the drying process should inhibit both electron conduction and Mg^2+^ ion diffusion to degrade electrochemical performances. To suppress such aggregation, graphene was used as an aggregation inhibitor and an electron conducting additive.^30,37^ The composite of MMO with graphene (MMO–G) was easily prepared by just adding graphene to the synthetic solution. The Mn K-edge XANES spectrum and the Mg/Mn rate of MMO–G were almost the same as those of MMO, indicating that graphene addition do not affect the structure of MMO (Fig. S1†). The SEM image and corresponding EDX mappings presented in Fig. 2c–f revealed that MMO particles were wrapped by wrinkled graphene to form larger MMO–G secondary particles. Thus, the aggregation of MMO particles could be suppressed by covering the particles with graphene.

**Fig. 3:**
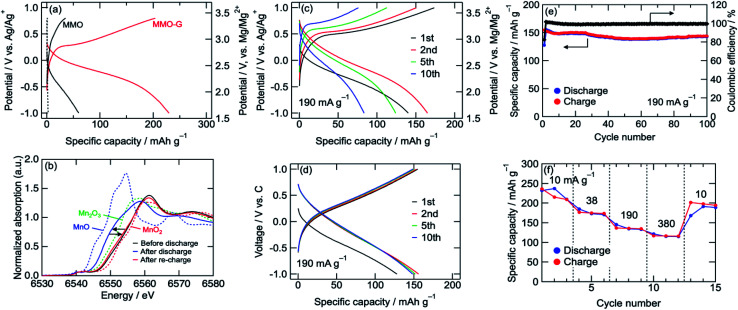
(a) Voltage curves of MMO and MMO–G cathodes, (b) Mn K-edge XANES spectra of MMO–G cathodes. (c) Voltage curves of MMO–G cathode at 190 mA g^−1^ using a 3-electrode cell or (d) a coin-type cell. (e) Cyclability and (f) rate-capability tests of MMO–G cathode using a coin-type cell.

The cathode performances of MMO electrodes with a 0.5 M Mg(ClO_4_)_2_/CH_3_CN electrolyte were investigated using a three-electrode cell at 25 °C with an Ag/Ag^+^ reference electrode (+2.6 V *vs.* Mg/Mg^2+^) and an activated carbon capacitor as a counter electrode (0.0 V *vs.* Ag/Ag^+^).^38^Fig. 3a shows the voltage curves of the MMO and MMO–G electrodes at a current density of 10 mA g^−1^. In the MMO electrode, no plateau was observed during discharge/charge and the discharge capacity was 60 mA h g^−1^, which was much smaller than that of an ideal one electron reaction per Mn (280 mA h g^−1^). This small capacity is possibly attributed to the slow diffusion of Mg^2+^ ions into large aggregates of MMO particles. On the other hand, the MMO–G electrode exhibited gentle slopes and a reversible capacity of more than 200 mA h g^−1^. Since the contribution of the electric double-layer capacitor (EDLC) of the graphene (dotted lines in Fig. 3a) is negligible, the enhancement of the specific capacity derives from the increase in the amount of the redox-active MMO particles. Moreover, d*Q*/d*V* curves of the MMO–G electrode exhibited a broad reductive peak at −0.2 V and a sharp oxidative peak at 0.3 V, supporting the redox of MMO (Fig. S2†). The obtained discharge capacity of 230 mA h g^−1^ corresponds to 0.83 electrons transferred per Mn, indicating that the average valence state of Mn changed from +3.19 to +2.36 and the discharge reaction can be expressed by the following equation:

3 Mg_0.81_Mn_2_O_4_ + 0.83Mg^2+^ + 1.66e^−^ → Mg_1.64_Mn_2_O_4_

Fig. 3b shows Mn K-edge XANES spectra of the MMO–G electrodes. The Mn K-edge was shifted to lower energies during discharge, and almost recovered after re-charge, indicating the reversible redox reaction of Mn. The Mn K-edge position at the full discharge (−1.0 V *vs.* Ag/Ag^+^) was approximately in the middle between those of MnO and Mn_2_O_3_, supporting eqn (3). According to the equation, it is indicated that both eqn (1) and (2) proceeded with a partial formation of rock-salt phase during the discharge.

A rate capability test of the MMO–G electrode was also carried out using the three-electrode cell. Fig. 3c shows voltage curves of an MMO–G electrode at a current density of 190 mA g^−1^. Even at a moderate current density, reversible discharge/charge proceeded at room temperature with little change in the voltage curves. However, the reversible capacity gradually decreased with the number of cycles, probably due to the dissolution of Mn to the electrolyte or the exfoliation of the electrode from the current collector during discharge/charge.

To reduce the amount of electrolyte and add a confining pressure on the electrode for suppressing the dissolution and exfoliation, a cycle test with a 2032 coin-type cell was investigated. Though the voltage curves with the coin cell slightly reflected the EDLC behaviour of the activated carbon counter electrode, the cell was well cycled more than 100 times without any large capacity loss (Fig. 3d and e). The Mn K-edge position of the electrode after 10th charge was almost the same as that after first charge, suggesting the redox of Mn proceeds at least 10th cycle without decomposition of MMO (Fig. S3†). Furthermore, the cell exhibited a relatively high rate capability (Fig. 3f) even at 380 mA g^−1^ (corresponding to a 1.4 C-rate) compared with previous reports (Table S1†), indicating that the Mg^2+^ intercalation/deintercalation proceeded rapidly due to small diffusion paths in the ultrasmall MMO particles covered by graphene prepared by the alcohol reduction method adopted here.

## Conclusions

Cubic spinel-type Mg–Mn oxide nanoparticles with sizes of approximately 5 nm and their composites with graphene as cover were successfully synthesized at room temperature and exhibited a moderate rate cathode performance at room temperature. Both downsizing the primary particles of active materials and suppressing the aggregation of particles are important for further development of cathodes in Mg-ion batteries. Especially in MgMn_2_O_4_ cathode, utilization of meta-stable cubic phase is important. The alcohol reduction process presented in this letter is a facile method to synthesize ultrasmall cathode materials.

## Experimental section

### Synthesis of Mg–Mn binary oxides

A precursor *n*-Bu_4_NMnO_4_ was synthesized according to a reported procedure.^30^ In brief, an aqueous *n*-Bu_4_NBr solution was slowly added to an aqueous KMnO_4_ solution under vigorous stirring, and the mixed solution was subsequently stirred for 1 h to obtain *n*-Bu_4_NMnO_4_. A solution of *n*-Bu_4_NMnO_4_ in acetonitrile (2.5 M, 1 mL) was slowly added to an anhydrous MgCl_2_ solution in ethanol (0.1 M, 100 mL) under vigorous stirring to form a brown colloidal solution. The solution was stirred for 30 min followed by addition of deionized water to form precipitates of the Mg–Mn binary oxide (MMO). The precipitates were collected by membrane filtration, washed with water and ethanol, and dried at 120 °C. A composite of MMO with graphene (MMO–G) was similarly obtained by dispersing 50 mg of graphene nanopowder (G-10, EM Japan Co., Ltd.) in the MgCl_2_ solution before adding the *n*-Bu_4_NMnO_4_ solution. Caution: *n*-Bu_4_NMnO_4_ can react violently with itself (the MnO_4_^−^ anion can oxidize the *n*-Bu_4_N^+^ cation) and possibly catch fire, hence it should be handled with appropriate care and stored under appropriate conditions (*e.g.* refrigerated conditions).

### Material characterization

Synchrotron powder X-ray diffraction (XRD) patterns with a wavelength of *λ* = 0.775 Å were collected at the BL5S2 beamline of the Aichi Synchrotron Radiation Center. Samples were charged into Lindeman glass capillaries of 0.5 mm diameter and measured with a rotating stage and a PILATUS 100K detector. The Rietveld refinement was performed using the RIETAN-FP program.^39^ Mn K-edge X-ray absorption spectroscopy (XAS) was carried out using the transmission method at the BL11S2 beamline of the Aichi Synchrotron Radiation Center. Samples were sealed in an Al-laminated packaging film and attached to a sample holder with Mn foil. Energy calibrations were carried out using the first peak of Mn foil (6539 eV) in a derivative spectrum. Electrode samples were washed with acetonitrile and dried in an Ar-filled glove box before sealing. X-ray absorption near edge structure (XANES) and extended X-ray absorption fine structure (EXAFS) were analyzed by the Athena and Artemis programs.^40^ For the EXAFS analysis, the *k*-range of the FT was 3–14 Å^−1^ with a Hanning window of 1 Å^−1^, and the radial distance range of the inverse FT was 1–3 Å. Elemental analyses were performed using inductively coupled plasma atomic emission spectroscopy (ICP-AES) on an Optima 3300XL (PerkinElmer) and a CHN analyzer (Micro Corder JM10, J-Science Lab Co., Ltd.). Scanning electron microscopy (SEM) and Transmission electron microscopy (TEM) images were obtained using JSM-7800F (JEOL) and EM-002B (Topcon), respectively. Brunauer–Emmett–Teller (BET) surface areas were measured by N_2_ adsorption at 77 K using BELSORP-mini (MicrotracBEL).

### Electrochemical measurements

MMO and MMO–G were mixed with acetylene black (AB; Denka Black, FX-35, Denka Co., Ltd.) and polytetrafluoroethylene (PTFE; Teflon, 6-J, DuPont-Mitsui Fluorochemicals Co., Ltd.) at a weight ratio of 60/30/10 and 80/10/10, respectively. In the MMO–G cathode, MMO/graphene/AB/PTFE = 68/12/10/10 by weight. These mixtures were cut into 8 mm diameter disks of typically 2.5 mg and pressed on an Al mesh current collector to serve as cathodes. For the anodes, a nanoporous activated carbon (Maxsorb®, MSC-30, Kansai Coke and Chemicals Co., Ltd.) was mixed with AB and PTFE at a weight ratio of 8/1/1 and typically 15 mg of the mixture was pressed on a SUS 304 stainless steel mesh current collector. The electrodes were dried at 160 °C under vacuum and introduced into an Ar-filled glove box. For the electrolyte solution, 0.5 M Mg(ClO_4_)_2_ (Sigma-Aldrich) dissolved in acetonitrile (Kanto Chemical Co., Inc.) was prepared and stored over molecular sieves. The cathode, the anode, and the electrolyte were assembled in a three-electrode cell (EC Frontier Co., Ltd.) with an Ag/Ag^+^ reference electrode or a 2032 coin-type cell (Hohsen Corp.) with a glass-fiber separator (GA-55, Toyo Roshi Kaisha, Ltd.). The amount of the electrolyte was 2 mL with the three-electrode cell and 0.1 mL with the coin-type cell, respectively. For the reference electrode, a double junction reference electrode was used with internal 0.01 M AgNO_3_ + 0.1 M *n*-Bu_4_NClO_4_ solution in acetonitrile separated by porous glasses. The aforementioned cell preparations were conducted in an Ar-filled glove box. Charge/discharge tests were carried out at 25 °C in constant-current (CC) mode using a multi-channel potentiostat system (VMP3, Bio-Logic Science Instruments) or a battery test system (HJ-1001SD8, Hokuto Denko Corp.). The specific capacity and the current density were calculated on the basis of the weight of MMO in the electrode. Caution: anhydrous perchlorate salts are potentially explosive and should be handled with appropriate care.

## Conflicts of interest

There are no conflicts to declare.

## Supplementary Material

RA-009-C9RA08626A-s001

## References

[cit1] Tarascon J.-M., Armand M. (2001). Nature.

[cit2] Bruce P. G., Freunberger S. A., Hardwick L. J., Tarascon J.-M. (2012). Nat. Mater..

[cit3] Black R., Adams B., Nazar L. F. (2012). Adv. Energy Mater..

[cit4] Ji X., Nazar L. F. (2010). J. Mater. Chem..

[cit5] Muldoon J., Bucur C. B., Gregory T. (2014). Chem. Rev..

[cit6] Muldoon J., Bucur C. B., Gregory T. (2017). Angew. Chem., Int. Ed..

[cit7] Bucur C. B., Gregory T., Oliver A. G., Muldoon J. (2015). J. Phys. Chem. Lett..

[cit8] Yoo H. D., Shterenberg I., Gofer Y., Gershinsky G., Pour N., Aurbach D. (2013). Energy Environ. Sci..

[cit9] Mohtadi R., Mizuno F. (2014). Beilstein J. Nanotechnol..

[cit10] Levi E., Levi M. D., Chasid O., Aurbach D. (2009). J. Electroceram..

[cit11] Mao M., Gao T., Hou S., Wang C. (2018). Chem. Soc. Rev..

[cit12] Okamoto S., Ichitsubo T., Kawaguchi T., Kumagai Y., Oba F., Yagi S., Shimokawa K., Goto N., Doi T., Matsubara E. (2015). Adv. Sci..

[cit13] Kurihara H., Yajima T., Suzuki S. (2008). Chem. Lett..

[cit14] Sinha N. N., Munichandraiah N. (2008). Electrochem. Solid-State Lett..

[cit15] Rahman M. F., Gerosa D. (2015). Optoelectron. Adv. Mater., Rapid Commun..

[cit16] Cabello M., Alcántara R., Nacimiento F., Ortiz G., Lavela P., Tirado J. L. (2015). CrystEngComm.

[cit17] Feng Z., Chen X., Qiao L., Lipson A. L., Fister T. T., Zeng L., Kim C., Yi T., Sa N., Proffit D. L., Burrell A. K., Cabana J., Ingram B. J., Biegalski M. D., Bedzyk M. J., Fenter P. (2015). ACS Appl. Mater. Interfaces.

[cit18] Yin J., Brady A. B., Takeuchi E. S., Marschilok A. C., Takeuchi K. J. (2017). Chem. Commun..

[cit19] Truong Q. D., Devaraju M. K., Tran P. D., Gambe Y., Nayuki K., Sasaki Y., Honma I. (2017). Chem. Mater..

[cit20] Tao S., Huang W., Liu Y., Chen S., Qian B., Song L. (2018). J. Mater. Chem. A.

[cit21] Liu G., Chi Q., Zhang Y., Chen Q., Zhang C., Zhu K., Cao D. (2018). Chem. Commun..

[cit22] Banu A., Sakunthala A., Thamilselvan M., Kumar P. S., Suresh K., Ashwini S. (2019). Ceram. Int..

[cit23] Zainol N. H., Hambali D., Osman Z., Kamarulzaman N., Rusdi R. (2019). Ionics.

[cit24] Ling C., Zhang R., Mizuno F. (2016). ACS Appl. Mater. Interfaces.

[cit25] Sinha A. P. B., Sanjana N. R., Biswas A. B. (1957). Acta Crystallogr..

[cit26] Barkhatov V. P., Balakirev V. F., Golikov Y. V., Kostitsin E. G. (1983). Phys. Status Solidi A.

[cit27] Mănăilă R., Păuşescu P. (1965). Phys. Status Solidi B.

[cit28] Rosenberg M., Nicolau P. (1964). Phys. Status Solidi B.

[cit29] Malavasi L., Tealdi C., Amboage M., Mozzati M. C., Flor G. (2005). Nucl. Instrum. Methods Phys. Res., Sect. B.

[cit30] Miyamoto Y., Kuroda Y., Uematsu T., Oshikawa H., Shibata N., Ikuhara Y., Suzuki K., Hibino M., Yamaguchi K., Mizuno N. (2015). Sci. Rep..

[cit31] Miyamoto Y., Kuroda Y., Uematsu T., Oshikawa H., Shibata N., Ikuhara Y., Suzuki K., Hibino M., Yamaguchi K., Mizuno N. (2016). ChemNanoMat.

[cit32] Nakai S., Uematsu T., Ogasawara Y., Suzuki K., Yamaguchi K., Mizuno N. (2018). ChemCatChem.

[cit33] Yamaguchi H., Yamada A., Uwe H. (1998). Phys. Rev. B.

[cit34] The XRD pattern was fitted using the cation mixing phase (Mg_0.8−2*x*_Mn_2*x*_)[Mg_*x*_Mn_1−*x*_]_2_O_4_ with *x* = 0.006(11), indicating the cation mixing of MMO is negligible

[cit35] The XRD pattern was not fitted successfully with (Mg_0.8_Mn_2*x*_)[Mn_1−*x*_]_2_O_4_

[cit36] Chen W., Zhan X., Luo B., Ou Z., Shih P.-C., Yao L., Pidaparthy S., Patra A., An H., Braun P. V., Stephens R. M., Yang H., Zuo J.-M., Chen Q. (2019). Nano Lett..

[cit37] Wang L., Vullum P. E., Asheim K., Wang X., Svensson A. M., Vullum-Bruer F. (2018). Nano Energy.

[cit38] Since acetonitrile-based electrolytes exhibit higher stability at high potential compared with commonly used ether-based electrolytes, the cathode performances were investigated using the acetonitrile-based electrolyte though the electrolyte does not exhibit Mg-deposition/dissolution at the Mg metal anode

[cit39] Izumi F., Momma K. (2007). Solid State Phenom..

[cit40] Ravel B., Newville M. (2005). J. Synchrotron Radiat..

