# Supplementary material for: Rapid room-temperature synthesis of ultrasmall cubic Mg–Mn spinel cathode materials for rechargeable Mg-ion batteries
Source: RSC Adv. 2019 Nov 8;9(62):36434–9. doi: 10.1039/c9ra08626a (PMC9075040; doi:10.1039/c9ra08626a)
Supplement: RA-009-C9RA08626A-s001 [file RA-009-C9RA08626A-s001.pdf]

Electronic Supplementary Material (ESI) for RSC Advances.
This journal is © The Royal Society of Chemistry 2019

Supplementary Information for

# Rapid room-temperature synthesis of ultrasmall cubic Mg–Mn spinel cathode materials for rechargeable Mg-ion battery

*Hiroaki Kobayashi,*[a] Kazuya Yamaguchi[b] and Itaru Honma[a]*

[a]Institute of Multidisciplinary Research for Advanced Materials, Tohoku University, 2-1-1 Katahira, Aoba-ku, Sendai, Miyagi, 980-8577, Japan.

[b]Department of Applied Chemistry, School of Engineering, The University of Tokyo, 7-3-1 Hongo, Bunkyo-ku, Tokyo, 113-8656, Japan.

*Corresponding author

Email: h.kobayashi@tohoku.ac.jp

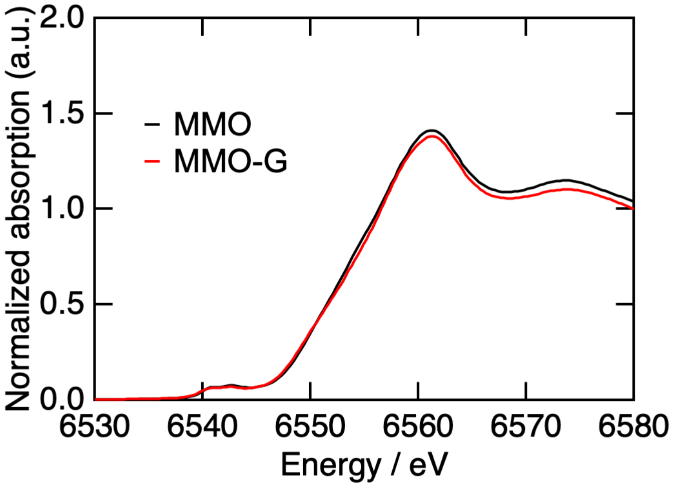


**Fig. S1.** Mn *K*-edge XANES spectra of MMO and MMO-G.

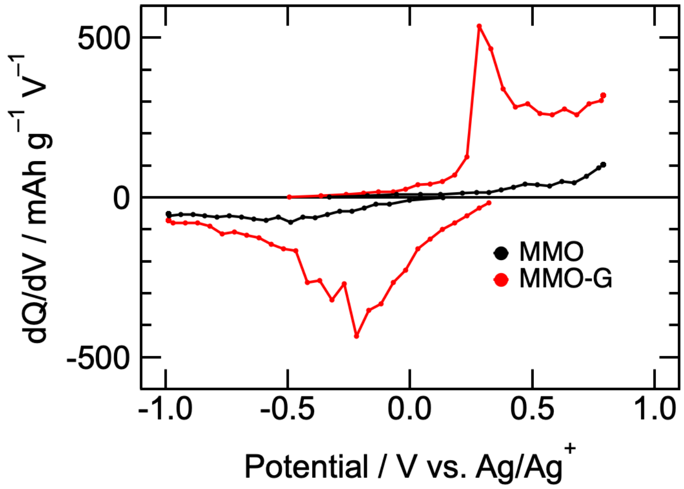


**Fig. S2.** dQ/dV curves of MMO and MMO-G cathodes.

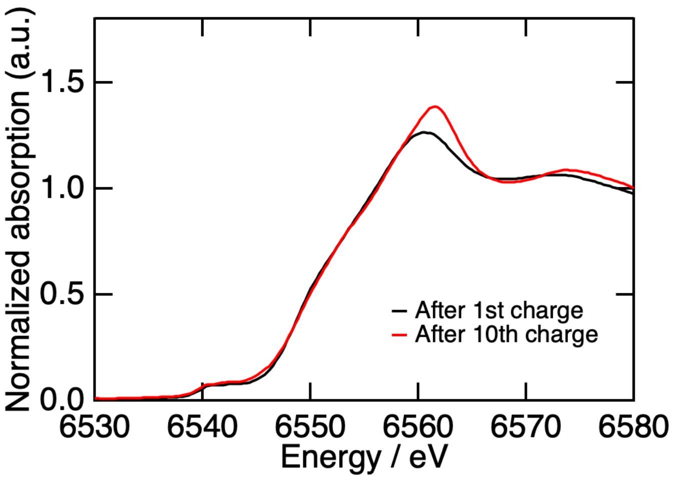


**Fig. S3.** Mn *K*-edge XANES spectra of MMO-G electrode after 1st and 10th charge.

**Table S1.** Comparison of the cathode performances for $MgMn_2O_4$ with anhydrous electrolytes.

| Procedure (Calcination condition) | Particle size | Electrolyte | Discharge capacity | Current density | Ref. |
|---|---|---|---|---|---|
| Coprecipitation (500 ºC, 24 h) | Not indicated | $Mg(TFSA)_2$/CsTFSA (150ºC) | 150 mAh $g^{-1}$<br>200 mAh $g^{-1}$ | 13.5 mA $g^{-1}$<br>5.4 mA $g^{-1}$ | [12] |
| Coprecipitation (750 ºC, 6 h) | 200 nm | 0.2 M $Mg(Ac)_2$ + 0.1 M $AlCl_3$/PC | 102 mAh $g^{-1}$ | Not indicated | [15] |
| Sol-gel (400 ºC, 10 h) | 17 nm | 0.5 M $Mg(ClO_4)_2$ /EC-DEC | 120 mAh $g^{-1}$ | 10 μA | [16] |
| Sol-gel (400 ºC, 10 h) | 11 nm | 0.5 M $Mg(TFSA)_2$ + 0.5 M DPGDME/AN | 220 mAh $g^{-1}$<br>80 mAh $g^{-1}$ | 27 mA $g^{-1}$<br>135 mA $g^{-1}$ | [18] |
| Sol-gel (550 ºC, 10 h) | 31 nm | 0.5 M $Mg(TFSA)_2$ + 0.5 M DPGDME/AN | 70 mAh $g^{-1}$<br>50 mAh $g^{-1}$ | 27 mA $g^{-1}$<br>135 mA $g^{-1}$ | [18] |
| Sol-gel (650 ºC, 12 h) | 40 nm | 0.5 M $Mg(ClO_4)_2$/AN | 100 mAh $g^{-1}$ | 4 mA $g^{-1}$ | [19] |
| Sol-gel (700 ºC, 24 h) | 120 nm | $Mg(TFSA)_2$/EC-PC | 225 mAh $g^{-1}$ | 1 mA $g^{-1}$ | [23] |
| Alcohol reduction (r.t., 30 min) | 5 nm | 0.5 M $Mg(ClO_4)_2$/AN | 230 mAh $g^{-1}$<br>150 mAh $g^{-1}$ | 10 mA $g^{-1}$<br>190 mA $g^{-1}$ | This work |
